# Combination of circulating tumor cell enumeration and tumor marker detection in predicting prognosis and treatment effect in metastatic castration-resistant prostate cancer

**DOI:** 10.18632/oncotarget.6167

**Published:** 2015-10-19

**Authors:** Kun Chang, Yun-Yi Kong, Bo Dai, Ding-Wei Ye, Yuan-Yuan Qu, Yue Wang, Zhong-Wei Jia, Gao-Xiang Li

**Affiliations:** ^1^ Department of Urology, Fudan University Shanghai Cancer Center, Fudan University, Shanghai, China; ^2^ Department of Oncology, Shanghai Medical College, Fudan University, Shanghai, China; ^3^ Department of Pathology, Fudan University Shanghai Cancer Center, Fudan University, Shanghai, China

**Keywords:** circulating tumor cell, epithelial-mesenchymal transition, metastatic castration-resistant prostate cancer, prognosis, stem cell

## Abstract

Although circulating tumor cell (CTC) enumeration in peripheral blood has already been validated as a reliable biomarker in predicting prognosis in metastatic castration-resistant prostate cancer (mCRPC), patients with favorable CTC counts (CTC < 5/7.5 ml) still experience various survival times. Assays that can reduce patients' risks are urgently needed. In this study, we set up a real-time quantitative polymerase chain reaction (RT-qPCR) method to detect epithelial-mesenchymal transition (EMT) and stem cell gene expression status in peripheral blood to validate whether they could complement CTC enumeration. From January 2013 to June 2014 we collected peripheral blood from 70 mCRPC patients and enumerated CTC in these blood samples using CellSearch system. At the same time, stem cell-related genes (ABCG2, PROM1 and PSCA) and EMT-related genes (TWIST1 and vimentin) were detected in these peripheral blood samples using an RT-qPCR assay. Patient overall survival (OS) and treatment methods were recorded in the follow-up. For patients who received first-line chemotherapy, docetaxel plus prednisone, PSA progression-free survival (PSA-PFS) and PSA response rate were recorded. At the time of analysis, 35 patients had died of prostate cancer with a median follow-up of 16.0 months. Unfavorable CTC enumerations (CTC ≥5/7.5 ml) were predictive of shorter OS (*p* = 0.01). Also, positive stem cell gene expression indicated poor prognosis in mCRPC patients (*p* = 0.01). However, EMT gene expression status failed to show any prognostic value in OS (*p* = 0.78). A multivariate analysis indicated that serum albumin (*p* = 0.04), ECOG performance status (p < 0.01), CTC enumeration (*p* = 0.02) and stem cell gene expression status (*p* = 0.01) were independent prognostic factors for OS. For the 40 patients categorized into the favorable CTC enumeration group, positive stem cell gene expression also suggested poor prognosis (*p* < 0.01). A combined prognostic model consisting of stem cell gene expression and CTC enumeration increased the concordance probability estimated value from 0.716 to 0.889 in comparison with CTC enumeration alone. For patients who received docetaxel plus prednisone as first-line chemotherapy, positive stem cell gene expression suggested a poor PSA-PFS (*p* = 0.01) and a low PSA response rate (*p* = 0.008). However, CTC enumeration and EMT gene expression status did not affect PSA-PFS or PSA response rates. As a result, detection of peripheral blood stem cell gene expression could complement CTC enumeration in predicting OS and docetaxel-based treatment effects in mCRPC patients.

## INTRODUCTION

In the past several years, great progress has been made in the management of metastatic castration-resistant prostate cancer (mCRPC). Several novel therapeutic agents including abiraterone, enzalutamide, cabazitaxel and radium-223 have demonstrated their life-prolonging efficacy in the treatment of mCRPC. However, the development of personalized and sequential management strategies has been hindered by the inability to identify distinct prognostic subgroups. Prostate-specific antigen (PSA), a commonly used prostate-specific biomarker, is not a reliable surrogate for survival end points because of insufficient sensitivity and specificity, and reliable prognostic and predictive biomarkers are urgently needed.

Recently, analysis of circulating tumor cells (CTCs) was shown to be of prognostic and predictive value in mCRPC [1-3]. Most of these studies used the CellSearch (Janssen Diagnostics, Raritan, NJ, USA) system for CTC analysis and found a consistent result, namely that an unfavorable CTC count (≥5 cells/7.5 mL) before therapy was associated with shorter overall survival (OS) than a favorable CTC count (< 5 cells/7.5 mL) in mCRPC patients [1, 2, 4]. Although CellSearch is the only US Food and Drug Administration (FDA)-cleared device for CTC detection and enumeration, it still has some limitations including a comparatively low detection rate and uncertainty of reliable finding in favorable counts. In this regard, although the survival times of patients with high CTC counts are uniformly poor, survival times for those with low CTC counts vary widely [5, 6]. These limitations may be due to the inability of the CellSearch system to detect CTCs that have undergone epithelial-mesenchymal transition (EMT), which results in lost expression of epithelial cell markers and re-expression of stem cell markers.

Therefore, we established a set of sensitive, highly reproducible, and fully standardized real-time quantitative polymerase chain reaction (RT-qPCR) assays to detect stem cell markers (ABCG2, PROM1 and PSCA) and EMT markers (TWIST1 and vimentin) in peripheral blood samples derived from mCRPC patients, and we further evaluated whether these markers could complement CellSearch CTC enumeration in predicting prognosis and treatment effect in mCRPC patients.

## RESULTS

### Patient characteristics

The clinicopathologic characteristics of our 70 mCRPC patients are summarized in Table 1. The median age was 70 years and the median PSA was 24.2 ng/ml at the time of inclusion. The site of metastasis included: bone (92.9%), lymph node (34.3%), liver (7.1%), lung (5.7%) and other soft tissue (7.1%). In all, 22 patients developed multi-organ metastasis at the time of the blood draw. After a median follow-up of 18 months (range, 12–26 months), 35 patients died of prostate cancer.

**Table 1:** Baseline clinicopathologic characteristics of 70 mCRPC patients

Characteristics	Median (Range)
Age, years	70 (55-85)
PSA, ng/ml	24.2 (2.6-269.6)
Hemoglobin, g/l	124 (69-156)
ALP, U/l	111 (47-2779)
LDH, U/l	208 (114-2467)
ALB, g/l	44.5 (32.3-52.2)
ECOG performance status, No. (%)	
0-1	63 (90.0)
≥2	7 (10.0)
Gleason score of primary lesion, No. (%)	
5-7	30 (42.9)
8-10	40 (57.1)
Site of metastatic disease^1^, No. (%)	
Bone	65 (92.9)
Lymph node	24 (34.3)
Liver	5 (7.1)
Lung	4 (5.7)
Other soft tissue	5 (7.1)

PSA, prostate specific antigen; ALP, alkaline phosphatase; LDH, lactate dehydrogenase; ALB, albumin; ECOG, Eastern Cooperative Oncology Group.

1 22 patients developed multi-organ metastasis.

### Detection of stem cell gene and EMT gene expression using RT-qPCR

Relative gene expression (RGE) was calculated using the 2^−ΔΔCt^ method (ΔΔCt = (Ct gene – Ct β-actin) patient sample − (Ct gene – Ct β-actin) healthy volunteer sample) [7, 8]. Baseline gene expression was precisely quantitated in 20 healthy male control subjects (Table 2). The median RGE for ABCG2, PROM1, TWIST1 and vimentin in mCRPC patients was 0.88, 1.38, 0.88 and 0.92, respectively. The cut-off RGE value was set at 1.78, 2.15, 1.89 and 2.75 for ABCG2, PROM1, TWIST1 and vimentin, respectively. As PSCA was not detected in healthy control subjects, PSCA was defined as positive if it could be detected within 40 cycles in RT-qPCR. The detection rate of ABCG2, PROM1, TWIST1, vimentin and PSCA in mCRPC patients is listed in Table 2. In total, 44.29% (31/70) of patients were stem cell gene expression positive as at least one of the three genes (ABCG2, PROM1 and/or PSCA) could be detected, and 47.14% (33/70) of patients were positive for EMT gene expression as at least one of the two markers (TWIST1 and/or vimentin) could be detected.

**Table 2:** RGE of multiple biomarkers in PBMCs of 70 mCRPC patients and 20 control subjects

	Control subjects	mCRPC patients
RGE of ABCG2		
Median (Minimum-Maximum)	0.88 (0.74-1.43)	0.91 (0.06-1289.86)
Cut-off^1^	1.78	1.78
No. of patient > cut-off	0	13
RGE of PROM1		
Median (Minimum-Maximum)	1.38 (0.54-2.08)	1.32 (0.01-222.51)
Cut-off^1^	2.15	2.15
No. of patient > cut-off	0	20
RGE of PSCA		
Median (Minimum-Maximum)	/	/
Cut-off^2^	Undetected	Detected in 5 patients
No. of patient > cut-off	0	5
RGE of TWIST1		
Median (Minimum-Maximum)	0.88 (0.74-1.32)	1.27 (0.01-1369.09)
Cut-off^1^	1.89	1.89
No. of patient > cut-off	0	27
RGE of Vimentin		
Median (Minimum-Maximum)	0.92 (0.54-2.08)	0.85 (0.03-40.96)
Cut-off^1^	2.75	2.75
No. of patient > cut-off	0	10

RGE, relative gene expression;

1 The cut-off was set for each gene marker at three standard deviation above the mean expression in healthy control subjects.

2 As PSCA was not detected in healthy control subjects, PSCA was defined as positive if it can be detected within 40 cycles in the RT-PCR.

### Prognostic value of stem cell, EMT gene expression status and CTC enumeration

**Figure 1:**
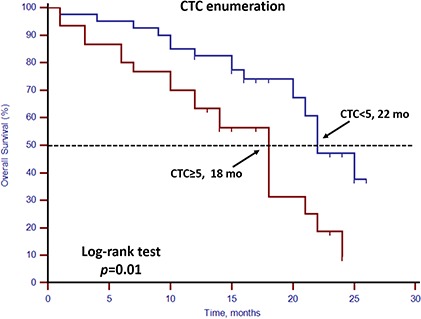
Kaplan–Meier estimates of OS in 70 mCRPC patients according to CTC enumeration Log-rank *p* = 0.01 (Cox HR: 2.44 (95% CI: 1.23–4.84)).

**Figure 2:**
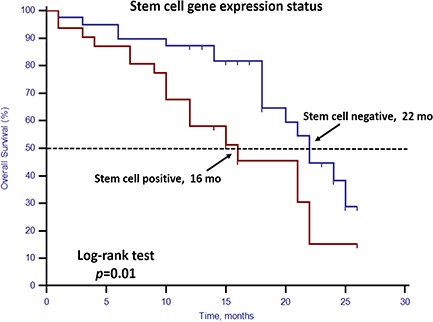
Kaplan–Meier estimates of OS in 70 mCRPC patients according to stem cell gene expression status Log-rank *p* = 0.01 (Cox HR: 2.43 (95% CI: 1.21–4.87)).

**Figure 3:**
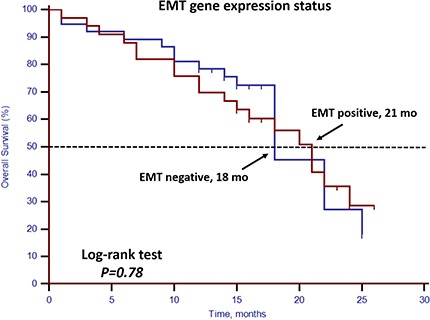
Kaplan–Meier estimates of OS in 70 mCRPC patients according to EMT gene expression status Log-rank *p* = 0.78 (Cox HR: 1.10, (95% CI: 0.56–2.15)).

Unfavorable CTC enumeration was detected (≥5 CTCs in 7.5 mL PB) in 30 patients (42.86%). Univariate analysis found that CTC enumeration and stem cell gene expression status were strong prognostic factors for OS (Table 3, Figures 1 and 2). However, EMT gene expression status had no prognostic value (Table 3, Figure 3). The median OS of patients with unfavorable CTC enumeration was significantly shorter than those with favorable CTC enumeration (18 vs. 22 months, *p* = 0.01, Figure 1). Moreover, the median OS of patients with stem cell gene expression was significantly shorter than those without stem cell gene expression (16 vs. 22 months, *p* = 0.01, Figure 2).

**Table 3:** Univariate and multivariate analysis of variables affecting overall survival in 70 mCRPC patients

Variables	Univariate analysis		Multivariate analysis	
	HR (95% CI)	*P* value	HR (95% CI)	*P* value
Age, years (continuous)	0.97 (0.93∼1.02)	0.25	-	-
PSA, ng/ml (continuous)	1.00 (1.00∼1.00)	0.08	-	-
LDH, U/l (continuous)	1.00 (1.00∼1.00)	0.07	-	-
Gleason score of primary lesion (≤8 vs >8)	1.09 (0.79-1.51)	0.58	-	-
Hemoglobin, g/l (continuous)	0.97 (0.95∼0.99)	<0.01	1.00 (0.97∼1.02)	0.69
ALP, U/l (continuous)	1.00 (1.00∼1.00)	<0.01	1.00 (1.00∼1.00)	0.08
ALB, g/l (continuous)	0.85 (0.79∼0.91)	<0.01	0.91 (0.83∼0.99)	0.04
ECOG performance status (0-1 vs ≥2)	5.50 (2.20-13.76)	<0.01	7.93 (2.35∼26.78)	<0.01
Chemotherapy (No vs Yes)	0.44 (0.22-0.89)	0.02	0.54 (0.21∼1.39)	0.20
CTC enumeration (<5 vs ≥5)	2.44 (1.23-4.84)	0.01	2.73 (1.21∼6.13)	0.02
Stem cell gene expression (negative vs positive)	2.43 (1.21-4.87)	0.01	3.18 (1.32∼7.65)	0.01
EMT gene expression (negative vs positive)	1.10 (0.56-2.15)	0.79		

PSA, prostate specific antigen; ALP, alkaline phosphatase; LDH, lactate dehydrogenase; ALB, albumin; ECOG, Eastern Cooperative Oncology Group; CTC, circulating tumor cell; EMT, epithelial mesenchymal transition.

### Stem cell gene expression in patients with favorable CTC enumeration

**Figure 4:**
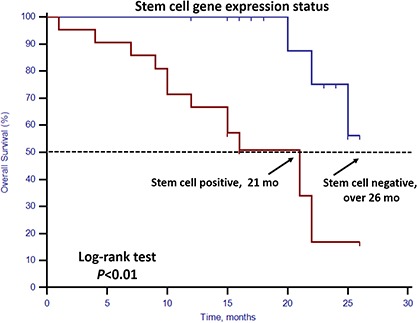
Kaplan–Meier estimates of OS in 40 mCRPC patients in the favorable CTC group according to stem cell gene expression status Log-rank *p* < 0.01 (Cox HR: 6.93, (95% CI: 1.90–25.31)).

Of the 40 patients with favorable CTC enumeration (CTCs < 5), 21 (52.50%) had detectable stem cell gene expression. Among them, those with and without stem cell gene expression differed significantly in OS (21 vs. before 26 months, respectively; p < 0.01, Figure 4).

### Univariate and multivariate analysis of variables affecting OS

In univariate analysis, hemoglobin, ALP, ALB, ECOG performance status, chemotherapy, CTC enumeration and stem cell expression status were significantly associated with shorter OS (Table 3). In multivariate analysis, only ALB, ECOG performance status, CTC enumeration and stem cell gene expression status were independent prognostic factors (Table 3).

### Combined prognostic model

**Figure 5:**
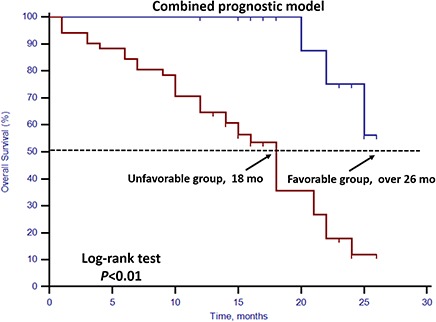
Kaplan–Meier estimates of OS in 70 mCRPC patients according to combined prognostic model Log-rank *p* < 0.01 (Cox HR: 7.65, (95% CI: 2.29–25.57)).

We developed a prognostic model using the combination of stem cell gene expression and CTC enumeration. In this model, the unfavorable group was defined as patients with either positive stem cell gene expression or unfavorable CTC enumeration (CTC ≥5), and the favorable group was defined as patients with both negative stem cell gene expression and favorable CTC enumeration. The statistical analysis showed a better concordance probability estimate (CPE) (0.889) in the combined prognostic model than CPE (0.716) in the model using CTC enumeration alone (Figure 5).

### Response to docetaxel-based first-line chemotherapy

**Figure 6:**
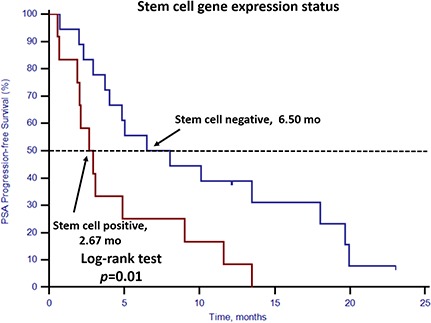
Kaplan–Meier estimates of PSA-PFS in 30 mCRPC patients who received docetaxel-based first-line chemotherapy according to stem cell gene expression status Log-rank *p* = 0.01 (Cox HR: 2.65, (95% CI: 1.18–5.95)).

**Figure 7:**
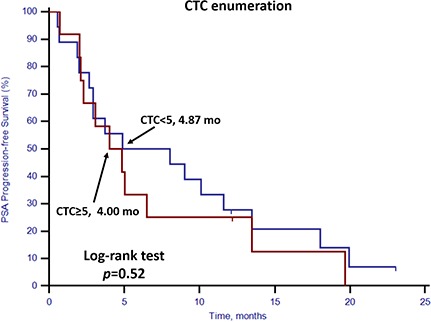
Kaplan–Meier estimates of PSA-PFS in 30 mCRPC patients who received docetaxel-based first-line chemotherapy according to CTC enumeration Log-rank *p* = 0.52 (Cox HR: 1.29, (95% CI: 0.59–2.82)).

**Figure 8:**
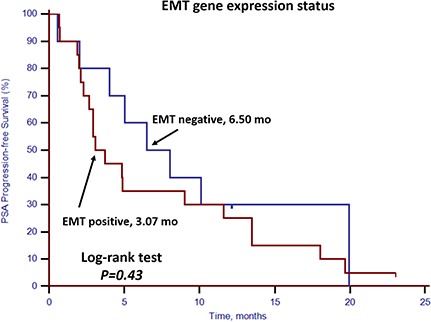
Kaplan–Meier estimates of PSA-PFS in 30 mCRPC patients who received docetaxel-based first-line chemotherapy according to EMT gene expression status Log-rank *p* = 0.43 (Cox HR: 1.40, (95% CI: 0.61–3.23)).

For the 30 patients who underwent docetaxel-based first-line chemotherapy, the PSA response rate and PSA progression-free survival (PSA-PFS) were evaluated. Patients with stem cell gene expression had a lower PSA response rate (25.0%, 3/12) compared with patients without stem cell gene expression (77.8%, 14/18; *p* = 0.008) (Table 4). Neither CTC enumeration nor EMT gene expression status showed any predictive value in determining PSA response rates (Table 4). PSA-PFS was shorter among patients with stem cell gene expression (*p* = 0.01, 2.67 vs. 6.50 months, Figure 6). However, CTC enumeration (p = 0.52) and EMT gene expression status (*p* = 0.43) did not show any prognostic value in PSA-PFS (Figures 7 and 8). For the remaining 40 patients who did not receive docetaxel-based first-line chemotherapy, neither stem cell gene expression status nor CTC enumeration nor EMT gene expression status had prognostic value.

**Table 4:** PSA responses rate of 30 patients treated by docetaxel based chemotherapy

Variables	Number of patients	PSA response, n (%)	*P* value
All patients	30	17 (56.7)	
CTC enumeration			
≥5	12	8 (66.7)	0.465
<5	18	9 (50.0)	
Stem cell gene expression			
Yes	12	3 (25.0)	0.008
No	18	14 (77.8)	
EMT gene expression			
Yes	20	11 (55.0)	1.000
No	10	6 (60.0)	

CTC, circulating tumor cell; EMT, epithelial mesenchymal transition.

## DISCUSSION

In this prospective study, we set up a highly sensitive RT-qPCR-based assay to detect stem cell gene and EMT gene expression in CTCs derived from mCRPC patients. This offers ease of collection and minimal onsite processing. More importantly, we found that both CTC enumeration and stem cell gene expression status were independent prognostic factors for OS in our mCRPC patients (Table 3, Figures 1 and 2). However, EMT gene expression status had no prognostic value (Figure 3). In addition, among the 40 patients with favorable CTC enumeration, those with positive stem cell gene expression experienced significantly shorter OS (Figure 4). Therefore, we established a prognostic model to predict the prognosis of CRPC patients by measuring the CPE using the combination of stem cell gene expression and CTC enumeration, which had a markedly improved risk assessment as shown in the combined prognostic model, which increased the CPE value from 0.716 to 0.889.

It is not surprising that CTC enumeration served as an independent prognostic factor for OS in our 70 mCRPC patients. The prognostic value of CTC enumeration using the CellSearch system in mCRPC patients has been reported previously. Goldkorn *et al.* reported that baseline high CTC counts enumerated using the CellSearch before therapy were associated with shorter median survival than low CTC counts in mCRPC patients treated with docetaxel [1]. Scher *et al.* also found that a biomarker panel containing the CTC number and LDH level was shown to be a surrogate for survival in mCRPC patients treated with abiraterone [2]. Interestingly, we also discovered that not all the patients in the favorable CTC group experienced a good prognosis, especially for the stem cell gene-positive ones. Because enumerating CTCs using the CellSearch system relies on the expression of epithelial markers, we may not be able to detect CTCs if they have undergone intrinsic modifications of their phenotype and lost their epithelial characteristics.

A critical concept that has emerged to explain the result is EMT. It enables epithelial cells to lose their apical–basal polarity, detach from neighboring cells, invade through the surrounding stroma, and become more resistant to apoptosis [9]. During this process, tumor cells lose expression of specific epithelial markers including E-cadherin [10], EpCAM [11], and cytokeratin [12], and gain expression of mesenchymal cytoskeletal and adhesion proteins such as vimentin and N-cadherin [13]. These proteins are thought to be controlled by a family of genes including Snail, Twist, and Zeb [14]. In addition, the process can trigger differentiated cells to acquire a multipotent cancer stem cell-like phenotype. This may mirror developmentally regulated EMT signaling pathways such as Wnt, Notch and Hedgehog, which drive cancer stem cell renewal and maintenance [15, 16].

Since most CTC detection methods rely on the capture of cells with antibodies specifically against the epithelial phenotype-specific markers EpCAM and cytokeratins, they may lead to relatively low detection rates. In our study, although the selected EMT markers did not show any prognostic value in predicting OS and response status to chemotherapy, stem cell gene expression status has proved to be a strong prognostic factor for OS. We also found an increased predictive power when combining CTC counts and stem cell gene expression status to discriminate the favorable from the unfavorable group with respect to OS.

In this study, we validated that detection of stem cell gene expression in peripheral blood can be predictive of the response status of docetaxel-based first-line chemotherapy for 30 mCRPC patients. Docetaxel at 75 mg/m^2^ once every 3 weeks with prednisone compared with mitoxantrone plus prednisone has been shown to confer a significant survival advantage in patients with mCRPC [17]. In China, docetaxel treatment has been established as standard chemotherapy for mCRPC patients. However, definitive prognostic factors at the initiation of docetaxel chemotherapy associated with disease progression and OS have not been identified.

PSA is not adequate to reflect the status of disease effectively. In 20% of mCRPC patients who finally respond to chemotherapy proven to prolong OS, an initial PSA increase is seen before the decline. The decline may not occur for up to 12 weeks or even longer [18-20]. Recently, the androgen receptor splice variant 7 (AR-V7) detected in CTCs from mCRPC patients was found to be a reliable marker associated with primary resistance to enzalutamide and abiraterone therapy. However, in follow-up studies, detection of AR-V7 in CTCs from mCRPC patients was proven to be unassociated with primary resistance to taxane chemotherapy [21].

The mechanisms of cancer stem cells in chemoresistance have long been investigated [22]. ABCG2, a member of ATP-binding cassette (ABC) transporters, is closely related to multi-drug resistance by reducing cytoplasmic chemotherapeutic drug concentrations [23]. Sanchez *et al.* proved that the pharmacological blockade and knockdown of ABC transporters could partially sensitize prostate cancer cells to chemotherapy [24]. Prominin-1, also known as CD133, has been well documented to be a putative cancer stem cell surface marker in a number of tumors including prostate cancer [25]. Richardson *et al.* found that in primary prostate cancer tissues, CD133+ cells exhibited characteristics of cancer stem cells including tumorsphere formation and the development of prostatic-like acini in SCID mice. [26]. Yang *et al.* also found that the CD133+ population of the C4-2B prostate cancer cell line was closely associated with docetaxel-based chemotherapy [27].

PSCA has been shown to be overexpressed in local as well as metastatic prostate cancer cells [28, 29]. Its expression level increases in cases of higher Gleason score, advanced tumor stage and progression of disease to an androgen-independent state [30, 31]. In our study, the detection of stem cell gene expression in the peripheral blood of mCRPC patients was associated with chemoresistance. This result suggested that these markers might act as therapeutic targets in the further treatment of prostate cancer.

The relatively small cohort size was one of the limitations of the current study. A validation cohort will be needed in any future study. Another limitation is that only 30 patients actually received docetaxel-based chemotherapy among the 70 patients recruited in this study. Although abiraterone, enzalutamide and cabazitaxel have been approved by the FDA for the treatment of mCRPC, they have not yet been marketed in China. As a result, patients in our cohort did not receive any of these new therapeutic agents during follow-up treatment, which probably contributed to the relatively short OS.

In summary, our study proved that the ABCG2, PROM1 and PSCA-based stem cell gene expression status could be used as a complement to CellSearch to predict OS. Furthermore, detection of these stem cell genes implicated the emergence of primary resistance to docetaxel-based first-line chemotherapy in mCRPC patients. Studies with larger cohorts will be needed to validate our preliminary findings.

## MATERIALS AND METHODS

### Prostate cancer cell line

LNCaP cells were cultured in RPMI1640 medium (GIBCO, Grand Island, NY, USA) containing 10% fetal bovine serum (FBS, GIBCO) at 37°C in a 5% CO_2_ atmosphere incubator.

### Patient selection and characteristics

From January 2013 to June 2014, 70 consecutive mCRPC patients (aged 55–85 years) treated at Fudan University Shanghai Cancer Center were included in this prospective study. No patient received any kind of chemotherapy before inclusion. At the time of inclusion, all patients underwent a complete evaluation including: physical examination, Eastern Cooperative Oncology Group (ECOG) performance status, routine clinical laboratory tests, measurement of serum PSA and testosterone levels, thoracic, abdominal and pelvic computed tomography (CT) scans, and a whole-body bone scan. Peripheral blood from each patient was collected after inclusion and before initiation of subsequent therapy. Because the China Food and Drug Administration (CFDA) have not approved abiraterone, enzalutamide and cabazitaxel, 30 of our 70 patients were treated with docetaxel plus prednisone as their first-line therapeutic regimen, and the remaining 40 patients who were intolerant to or refused chemotherapy were treated with ketoconazole, estrogen or traditional Chinese medicine. All patients were closely followed after inclusion. During follow-up, PSA levels were measured once a month in each patient to evaluate the PSA response, best PSA response, and PSA-PFS. According to the PCWG2 criteria, a PSA response referred to a ≥50% decline in PSA levels from baseline and maintained for ≥4 weeks; a best PSA response referred to the maximal percentage decrease in PSA levels from baseline; and PSA progression referred to an increase in PSA levels of 25% or more above the nadir (and by ≥2 ng/ml), with confirmation ≥4 weeks later [32, 33].

To define baseline expression in peripheral blood of the multiple mRNA markers used in this study, peripheral blood from control subjects was collected. We included 20 healthy male volunteers (aged 35–72 years, with no evidence of malignancy) as control subjects. This study was carried out in accordance with the ethical standards of the Helsinki Declaration II and approved by the Ethics and Scientific Committee of our institution. Written informed consent was obtained from all patients and healthy volunteers before any study-specific investigation was carried out.

### Blood sample collection

To reduce blood contamination by epithelial cells from the skin, the first 2 ml of blood was discarded and the collection tube was disconnected before withdrawing the needle at the end of the procedure. From each patient and healthy volunteer, 17.5 ml of peripheral blood was collected. The first 7.5 ml of blood was stored in a CellSave tube for CTC isolation and enumeration using the CellSearch^TM^ system (Veridex LLC, Warren, NJ, USA). The remaining 10 ml of blood was stored in an EDTA tube and immediately (within 2 hours) transferred to the laboratory and processed using Ficoll density gradient centrifugation (Ficoll-Paque Plus, TBDscience, China) to isolate peripheral blood mononuclear cells (PBMCs). The separated PBMCs, which contained CTCs, were stored at −80°C until RNA extraction.

### Enumeration of CTCs using the CellSearch^TM^ system

Isolation and enumeration of CTCs were performed at the central laboratory of our institution using the FDA-approved CellSearch^TM^ system. The process has been previously described in detail [3, 34]. Unfavorable CTC enumeration was defined as ≥5 CTCs in 7.5 ml of peripheral blood [3, 34].

### RNA isolation and cDNA synthesis

Total RNA was extracted from the separated PBMCs from 70 mCRPC patients and 20 healthy male volunteers using the TRIzol Reagent (Invitrogen, USA). RNA samples extracted from LNCaP cells served as a positive control. All RNA preparation and handling procedures were performed under RNase-free conditions in a laminar flow hood. Optical density measurements at 260 and 280 nm were used to quantify and assess the purity of the RNA. Reverse transcription of RNA was carried out with the PrimeScript RT Master Mix system (TaKaRa, Dalian, China). Complementary DNA was synthesized from 2 μg of total RNA isolated from PBMCs in a total volume of 20 μl, according to the manufacturer's instructions.

### Cancer stem cell gene and EMT gene expression analysis using RT-qPCR

RT-qPCR was performed using the 7900HT Fast RT-PCR System (Applied Biosystems, USA). The β-actin gene was used to normalize gene expression levels. Probes including ABCG2 (catalog no. Hs01053790_m1), PROM1 (catalog no. Hs01009250_m1), PSCA (catalog no. Hs04177224_g1), TWIST1 (catalog no. Hs01675818_s1), vimentin (catalog no. Hs00185584_m1) and β-actin (catalog no. Hs01060665_m1) were purchased from ABI Applied Biosystems. Relative gene expression (RGE) was calculated using the 2^−ΔΔCt^ method (ΔΔCt = (Ct gene – Ct β-actin) patient sample − (Ct gene – Ct β-actin) healthy volunteer sample) [7, 8]. RT-qPCR positivity was defined as gene expression greater than the cut-off threshold, which was set for each gene marker at three standard deviations above the mean expression in healthy control blood samples [8]. Stem cell gene expression was classified as positive if expression of one of the three stem cell genes (ABCG2, PROM1, and PSCA) was detected and EMT gene expression was classified as positive if expression of one of the two EMT genes (TWIST1 and vimentin) was detected.

### Statistical analysis

OS and PSA-PFS were calculated using the Kaplan–Meier method with the log-rank test to assess differences between groups. Univariate and multivariate analyses of prognostic factors were conducted using the Cox proportional hazards model. Because of the limited number of patients, only the variables that appeared to have a significant impact on OS by univariate analysis were included in the multivariate analysis. To evaluate the PSA response rate according to CTC enumeration, stem cell and EMT gene expression status, Fisher's exact test was used.

Prognostic significance of CTC enumeration and the combined prognostic model were assessed using the concordance probability estimate (CPE). The CPE measures the level of concordance between the survival time and the prognostic index, based on the linear combination of covariates in the Cox model, to provide an unbiased assessment of discrimination with survival data [6, 34]. CPE values range from 0.5–1.0, with 1.0 representing perfect concordance between the prognostic index and survival time [6, 34]. All statistical tests were done using SPSS, version 20 (SPSS Inc., Chicago, IL, USA) and R statistics package, version 2.8.1 (http://www.r-project.org/). A *P*-value < 0.05 was considered to indicate statistical significance.
